# A RBO na Web of Science: Qualidade reconhecida internacionalmente

**DOI:** 10.1055/s-0045-1812091

**Published:** 2025-11-18

**Authors:** Geraldo Motta

**Affiliations:** 1 Sociedade Brasileira de Ortopedia e Traumatologia, São Paulo, SP, Brasil

É com grande satisfação que anunciamos a indexação da Revista Brasileira de Ortopedia (RBO) na base *Web of Science* , mantida pela *Clarivate Analytics* . Este feito representa o reconhecimento internacional da qualidade editorial do periódico e consolida a posição da RBO como referência para a Ortopedia e Traumatologia na América Latina.

A inclusão na *Web of Science* não é um objetivo pontual, mas parte de uma estratégia editorial baseada em critérios rigorosos: revisão por pares transparente, pontualidade, integridade ética, internacionalização e relevância científica. Esses princípios orientaram todas as ações que nos trouxeram até aqui e continuarão norteando nosso trabalho.

Esse avanço não pertence apenas à RBO, mas a toda a comunidade ortopédica brasileira. Ao atingir essa posição, ampliamos a visibilidade das pesquisas nacionais, fortalecemos nossa capacidade de atrair colaborações internacionais e contribuímos para a projeção global da produção científica brasileira.

A responsabilidade, entretanto, cresce junto com o reconhecimento. É necessário manter a qualidade, estimular estudos originais de impacto, reduzir prazos editoriais e assegurar a constante atualização de nossas práticas.

Reafirmamos nosso compromisso com a excelência e convidamos todos a seguirem contribuindo para que a RBO, agora reconhecida em uma das mais importantes bases do mundo, continue sendo um espaço de inovação, credibilidade e impacto para a Ortopedia.

